# Supplementary material for: Cardiomyocyte gene programs encoding morphological and functional signatures in cardiac hypertrophy and failure
Source: Nat Commun. 2018 Oct 30;9:4435. doi: 10.1038/s41467-018-06639-7 (PMC6207673; doi:10.1038/s41467-018-06639-7)
Supplement: Supplementary file 1 — Supplementary Information [file 41467_2018_6639_MOESM1_ESM.pdf]

Supplementary Figures

Cardiomyocyte gene programs encoding morphological and functional signatures in cardiac hypertrophy and failure

Nomura et al.

Supplementary Figure 1

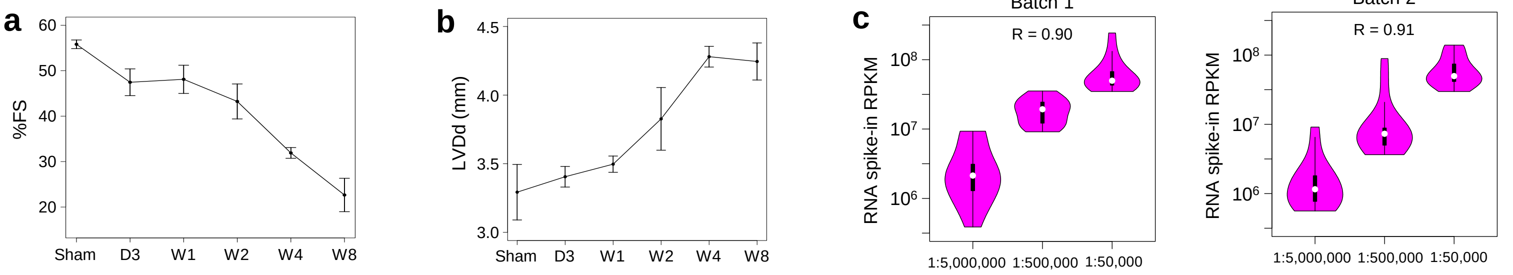

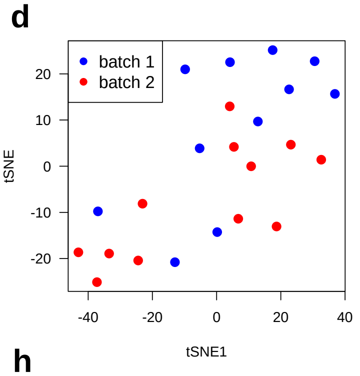

e

Number of cells	Sham	D3	W1	W2	W4	W8
cDNA library synthesis (N=540)	94	70	94	94	94	94
Passed the real-time PCR filter (N=482)	88	69	83	82	73	87
Passed the RPKM filter (N=396)	64	58	82	61	73	58

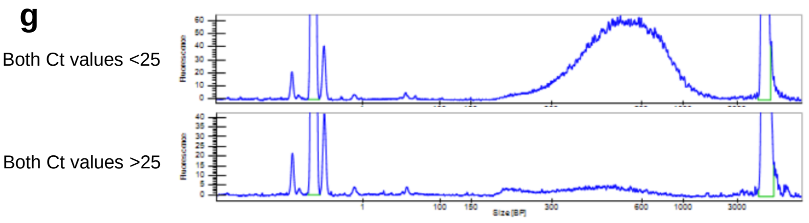

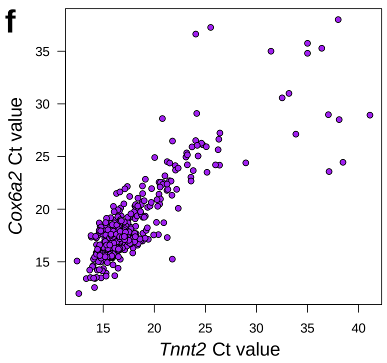

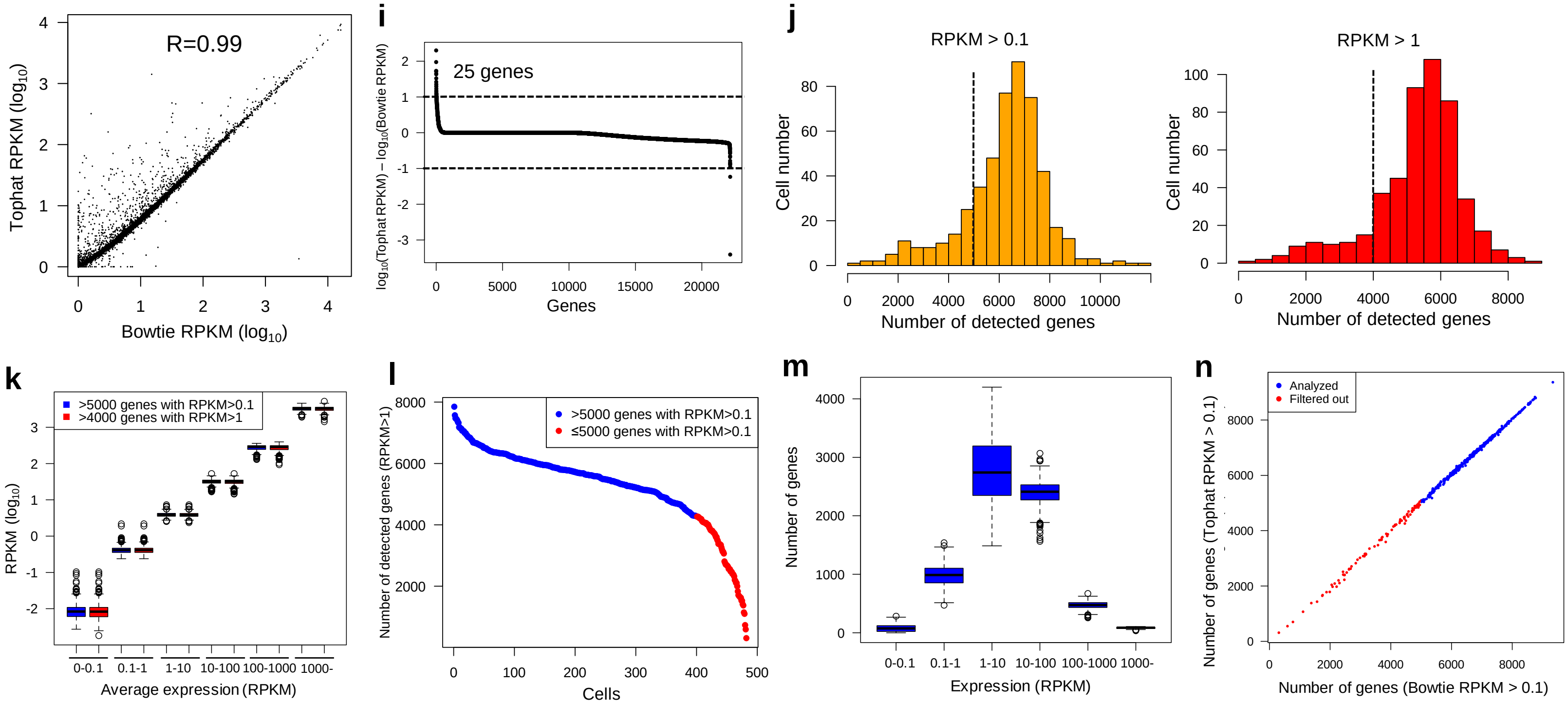

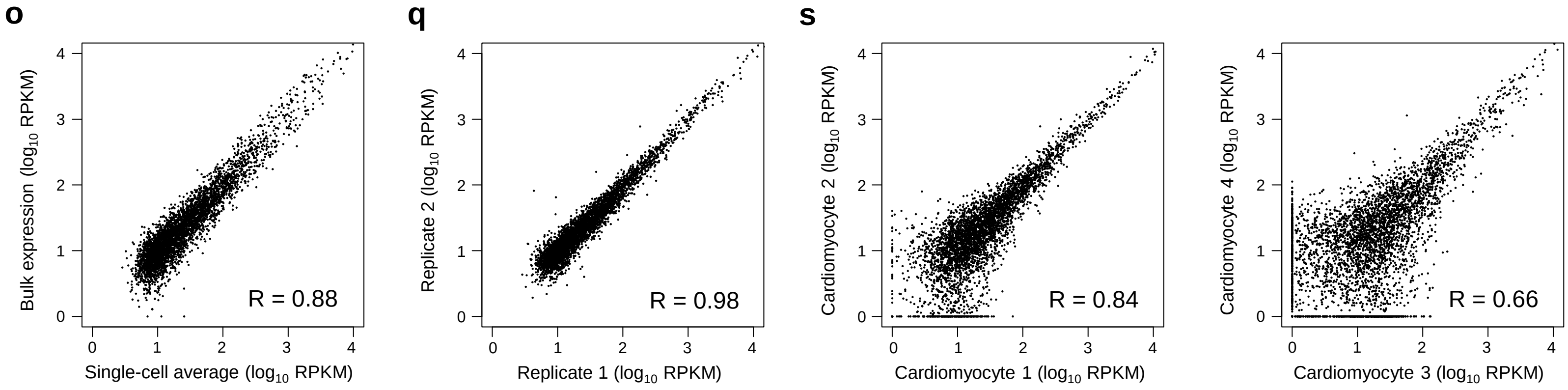

p Correlation between single-cell average and bulk expression

sample	Sham	D3	W1	W2	W4	W8
replicate1	0.96	0.99	0.79	0.85	0.88	0.90
replicate2	0.96	0.96	0.91	0.87	0.96	0.95

r Replicate correlation

Sham	D3	W1	W2	W4	W8
0.98	0.89	0.97	0.99	0.98	0.85

(legend on next page)

Supplementary Figure 1. Single-cardiomyocyte RNA-seq profiles.

(**a**,**b**) Echocardiographic assessment of the mice analyzed in this study. Line graphs show fractional shortening (%FS) (**a**) and left ventricular diastolic diameter (LVDd) (**b**). Mean and standard error of the mean are shown (*n* = 3–4 at each time point).

(**c**) Violin plot showing the expression levels of RNA spikes (RPKM) at the indicated concentrations (*n* = 15 at each concentration). A good correlation between the RPKM values and concentrations supports the accuracy of our single-cell RNA-seq analysis.

(**d**) t-SNE plot using single-cardiomyocyte transcriptomes from normal C57BL/6 mice (RPKM values) in 2 different batches. Cardiomyocytes from different batches could not be classified.

(**e**) Table showing the number of samples during the filtering steps.

(**f**) Scatter plot showing the Ct values (*Tnnt2* and *Cox6a2*) of single-cardiomyocyte cDNA libraries (*n* = 540).

(**g**) Representative results of capillary electrophoresis to assess the efficacy of the reverse transcription and amplification steps.

(**h**) Scatter plot showing the correlation between averaged single-cell RPKM values of sham cardiomyocytes mapped by Tophat and Bowtie.

(**i**) Plot showing the differences between averaged single-cell RPKM values of sham cardiomyocytes mapped by Tophat and Bowtie. There were 25 genes that were identified to be 10-fold higher in Tophat mapping RPKM values. Gene ontology analysis revealed that ribosomal protein genes were enriched in these genes (*p* = 7.9e-6).

(**j**) Bar plots showing the distribution of the number of detected genes with RPKM > 0.1 (left) and RPKM > 1 (right) across all cardiomyocytes (*n* = 482). More than 5,000 genes were detected with RPKM > 0.1 in 82.1% of cardiomyocytes (396/482 cells), and more than 4,000 genes were detected with RPKM > 1 in 86.9% of cardiomyocytes (419/482 cells).

(**k**) Boxplot showing the averaged expression levels of all cardiomyocytes (*n* = 482). Genes are categorized by the expression levels. Expression levels were comparable between cardiomyocytes that had more than 5,000 genes with RPKM > 0.1 (*n* = 396) and those that had more than 4,000 genes with RPKM > 1 (*n* = 419). Horizontal lines indicate the medians. Boxes show the 25th–75th percentiles. Whiskers represent the minimum and maximum values.

(**l**) Plot showing the number of detected genes (RPKM > 1). Among all of the cardiomyocytes that had more than 5,000 genes with RPKM > 0.1 (*n* = 396), over 4,000 genes were detected with RPKM > 1. Cells are ordered by the number of detected genes.

(**m**) Boxplot showing the number of genes in cardiomyocytes that had more than 5,000 genes with RPKM > 0.1 (*n* = 396). Genes are categorized by the expression levels. Horizontal lines indicate the medians. Boxes show the 25th–75th percentiles. Whiskers represent the minimum and maximum values.

(**n**) Scatter plot showing the numbers of detected genes (RPKM > 0.1) mapped by Tophat and Bowtie. The difference of mapping tools does not affect the number of detected genes both in cardiomyocytes analyzed in this study (*n* = 396, blue) and in those filtered out through the quality control process (*n* = 86, red).

(**o**) Scatter plot showing the correlation between the single-cell average ($\log_{10}$ RPKM) and bulk expression ($\log_{10}$ RPKM) in cardiomyocytes from mice at 4 weeks after pressure overload (replicate 1).

(**p**) Table showing the correlations between the single-cell average and bulk expression at each time point.

(**q**) Scatter plot showing the correlation between biological replicates of single-cell average transcriptomes from mice at 4 weeks after pressure overload.

(**r**) Table showing the correlations between biological replicates of single-cell average transcriptomes at each time point.

(**s**) Representative scatter plots showing the correlations of single-cell transcriptomes between cardiomyocytes from the same mice (4 weeks after pressure overload). Relationships between cardiomyocytes in the same clusters (left) and in the different clusters (right) are shown.

Supplementary Figure 2

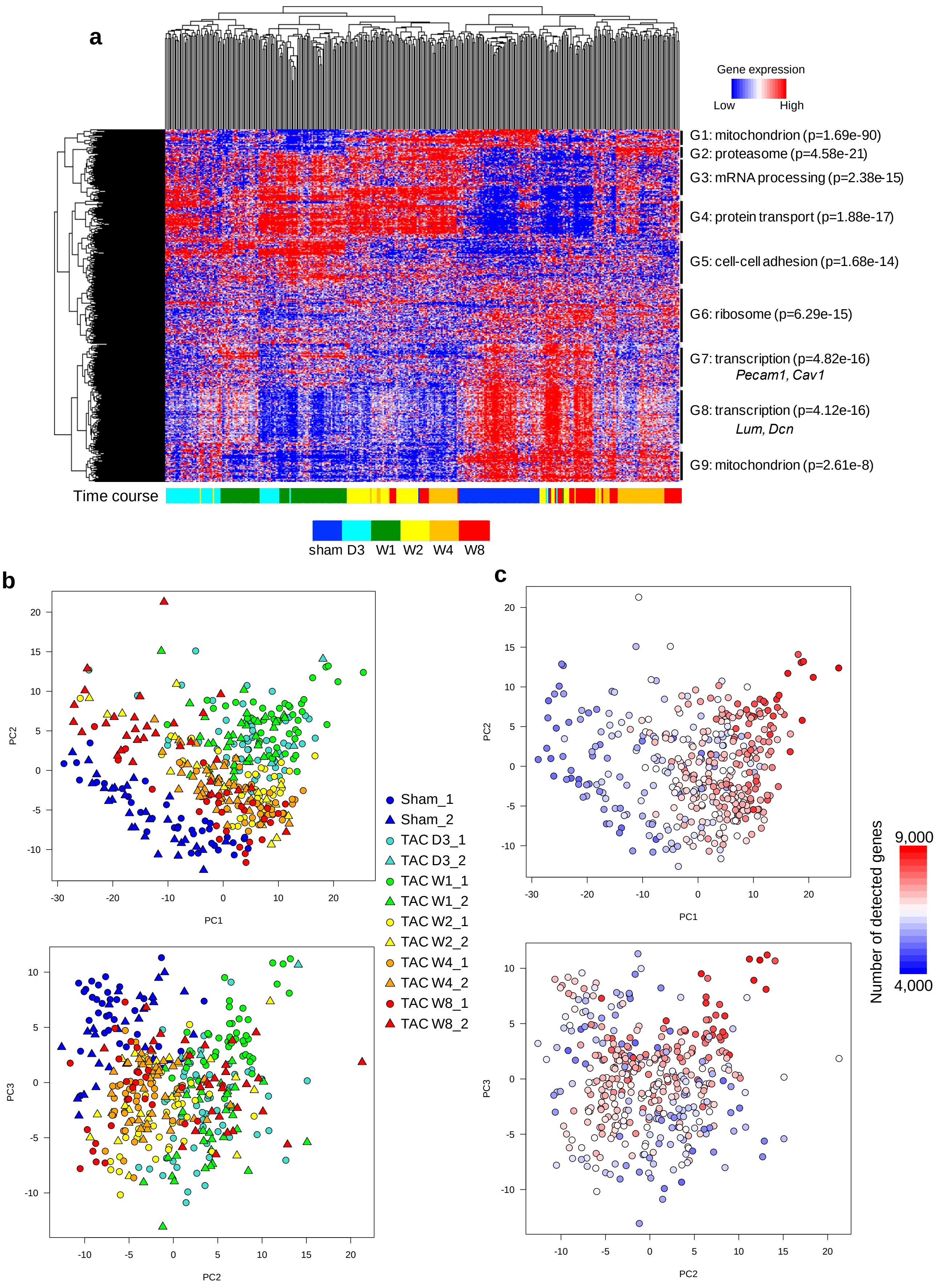

Supplementary Figure 2. Global landscape of single-cardiomyocyte RNA-seq.
(**a**) Hierarchical clustering of all cardiomyocytes (*n* = 396) using all genes (*n* = 10,720) expressed (RPKM ≥ 5) in at least 1% of cardiomyocytes.
(**b**,**c**) PCA plot colored by batches (**b**) and by the number of detected genes (**c**).

Supplementary Figure 3

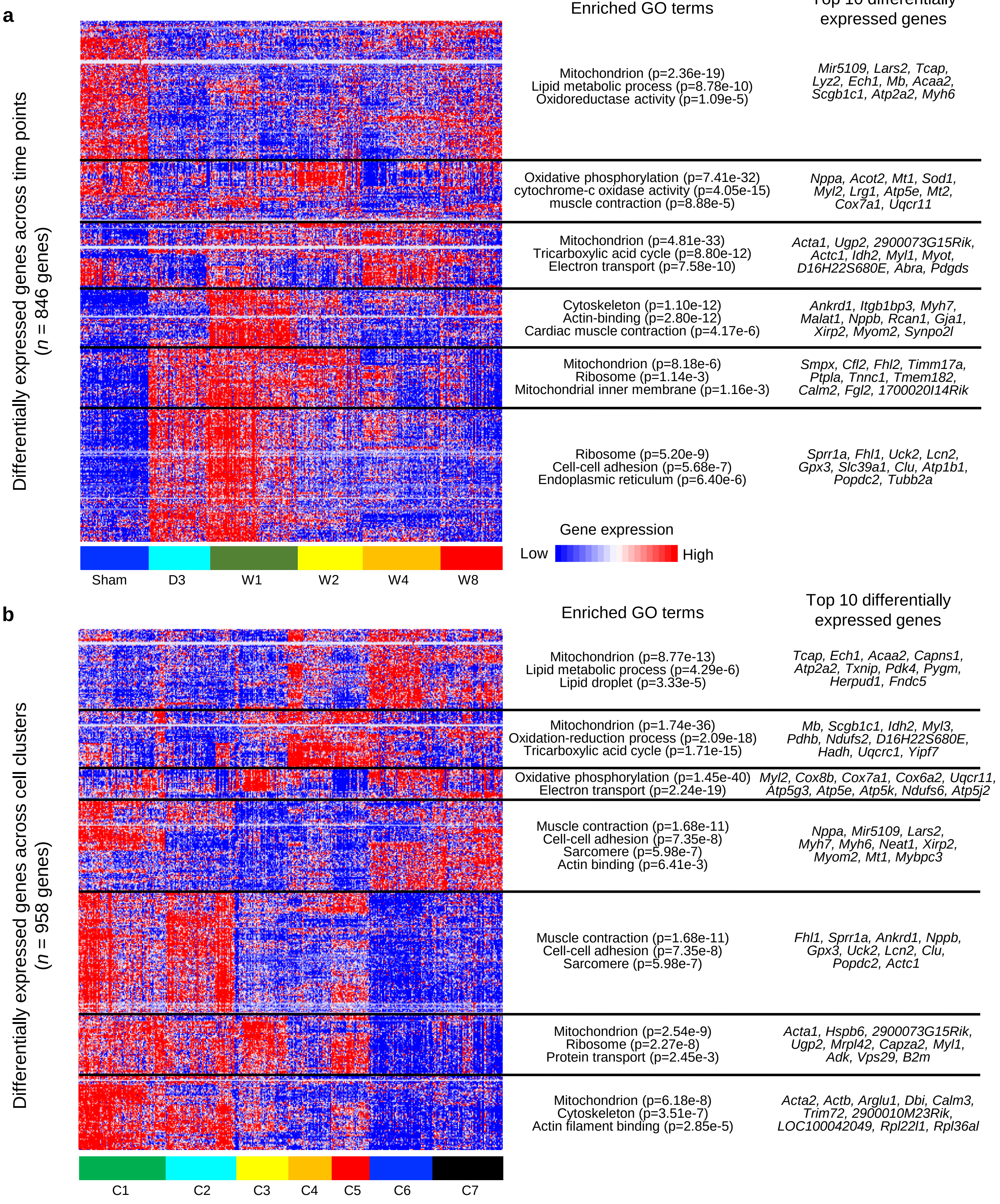

Supplementary Figure 3. Differential expression analysis.
(**a**,**b**) Heatmap showing the expression levels of genes differentially expressed across the time points (**a**) and cell clusters (**b**). Top 10 differentially expressed genes are also denoted.

Supplementary Figure 4

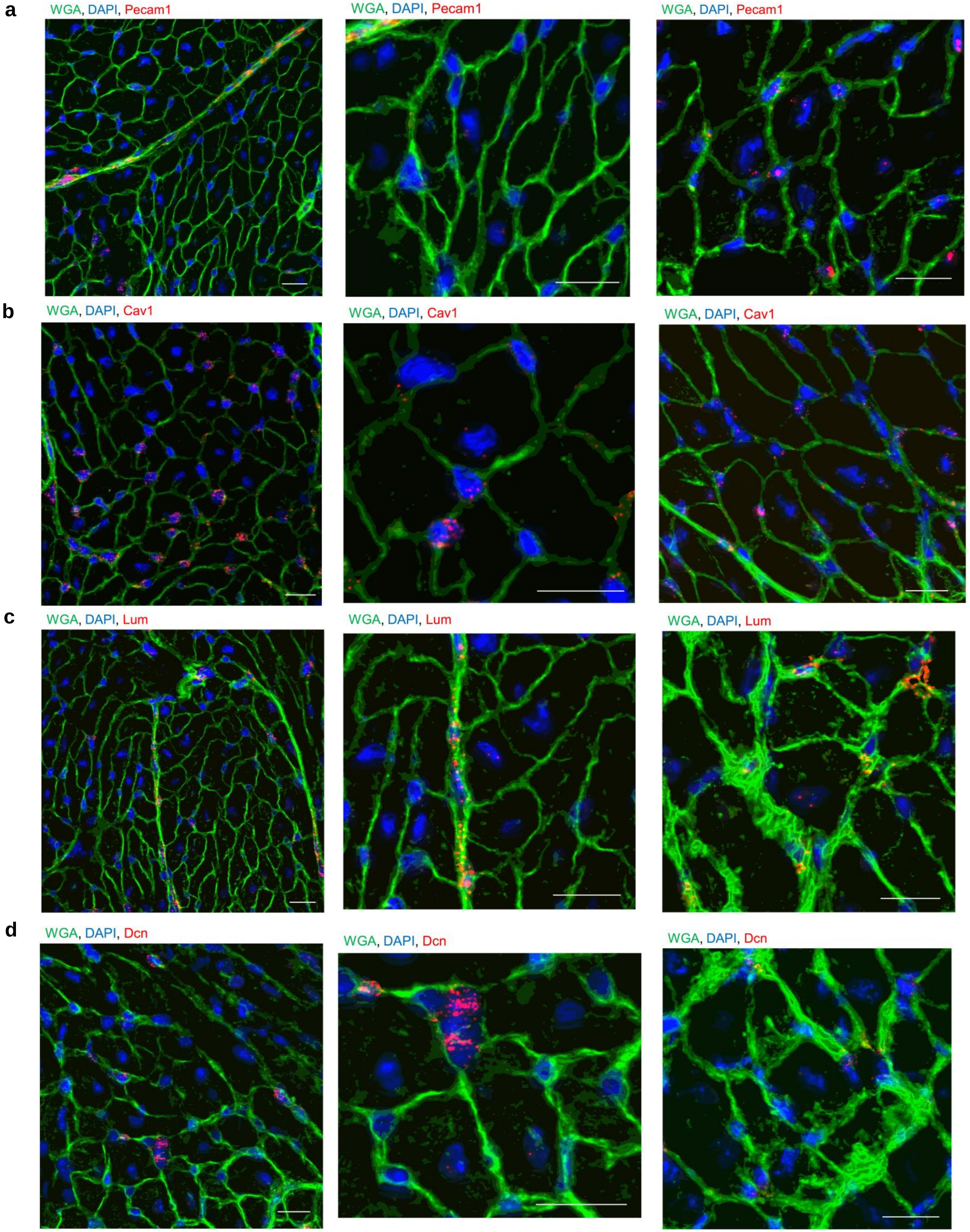

Supplementary Figure 4. Single-molecule RNA *in situ* hybridization of endothelial and fibrotic genes.
(**a**-**d**) Single-molecule RNA *in situ* hybridization of endothelial and fibrotic genes **(a**, *Pecam1*; **b**, *Cav1*; **c**, *Lum*; **d**, *Dcn*) in the heart from mice after sham (left and middle) and TAC (right) operation (week 8). Scale bar, 20 μm.

Supplementary Figure 5

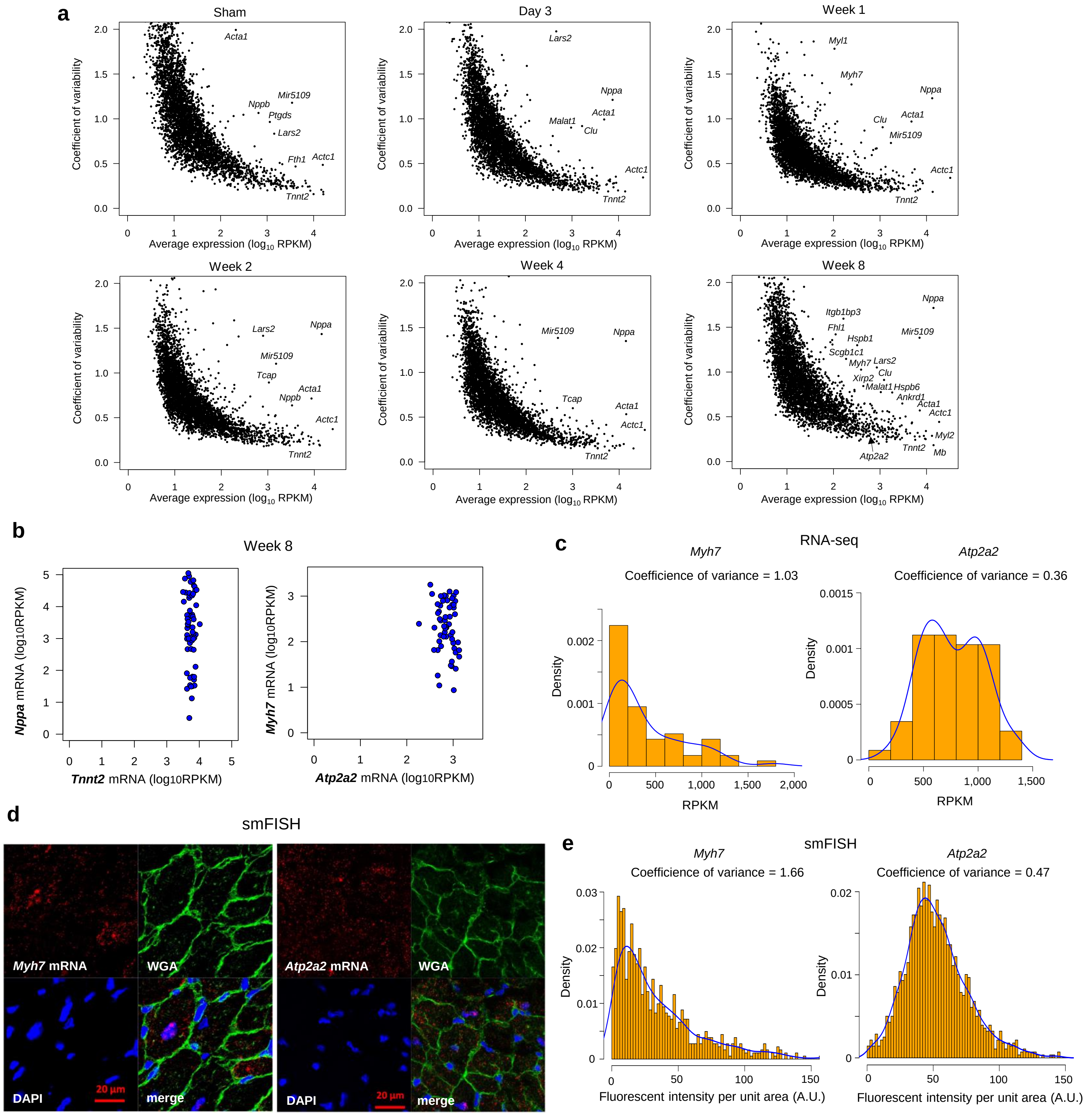

Supplementary Figure 5. Variability of gene expression in cardiomyocytes.
(**a**) Scatter plots showing the variability of gene expression in cardiomyocytes from mice at each time point.
(**b**) Scatter plots showing the single-cell expression values of *Nppa* and *Tnnt2* (left) and *Myh7* and *Atpa2a2* (right) in cardiomyocytes from mice at 8 weeks after pressure overload.
(**c**) Bar plots showing the distribution of cells corresponding to the single-cell expression level (RPKM) detected by single-cardiomyocyte RNA-seq at 8 weeks after pressure overload. Density plots are also shown with blue curves.
(**d**) Representative images of smFISH of the heart from mice at 8 weeks after pressure overload. Wheat germ agglutinin (WGA) and DAPI are used as markers of the plasma membrane and nucleus, respectively. Scale bar, 20 µm.
(**e**) Bar plots showing the distribution of cells corresponding to the single-cell fluorescent intensity detected by smFISH of the heart at 8 weeks after pressure overload. Density plots are also shown with blue curves.

Supplementary Figure 6

a

Tophat module

Bowtie module

Module	1	10	11	12	13	14	15	16	17	18	19	2	20	21	22	23	24	3	4	5	6	7	8	9
1	0.349	318	0	0	0	0	0	0	0	0.941	0	0	0	0	0	0	0	0	65.68	0	0	0	20.49	0
2	0.251	500	0	0	0.011	0	0	0.026	0	0.029	0	0	0	0	0	0	0	0	0	0	0	0	0.175	0
3	0	0	0.009	0.292	0.366	0	0	0.028	0	0.031	0	0	7.99	78.69	0	0	0	0	0.187	2.32	0	0	0.066	0
4	0	0	0	0	0	0	0	0	0	0	0.33	2.425	0.001	0	0.166	0.166	0	0	0	0	500	0	0	0
5	0	0	0	0	0	0	0	0	0	0	0	0	0.007	6.083	0	0	0	0	0.13	0	0	0	115.5	0
6	0	0	0	0	0	0	0	0	0	0	0	0	0.008	0	0	0	0	0	0	241.1	0	0.043	0	0
7	0	0	0	0	0	0	0	0	0	0.18	0	0	0	0	0	0	0	0	0	0	0	278.3	0	0
8	0	0	0	0	0	0.973	0.518	0	0	0	0.566	0	0.514	0	0	0	0	0	0	0.086	0	0	0.068	0.643
9	0.435	0	0	0	0.13	0.165	0	5.866	0.525	0	0	0	0.299	0	94.62	0	0.342	0	0	0.019	0	0	0	0.091
10	0	0	0	0	0	0	0	0	0	0	0	0	185.8	0	0	0	0	0	0	0	0	0	0	0
11	0	4.975	0	0	0	0	0	0	0	65.78	0	0	0	0	0	0	0	0	7.225	0	0	0	0	0.76
12	0	0.028	0	0	132.6	0	0	0.211	0	0.22	0	0	0	0	0	0	0	0	0	0	0	0	0	0
13	0	0	0	0.145	0.169	0.208	0	0.22	0.633	0.229	0.678	0	0.224	0	0	0	0.4	0	0	0.032	0	0	0	0.124
14	0	0	0	0.451	0	156.1	0	0	0.22	0	0	0	0.224	0	0	0	0	0	0.023	0.584	0	0	0	0
15	0	0	0	0	0	0	0	0	0	0	0	0	0.228	0.262	0	0	0	0	0	0	0	0	0	0
16	0	0	0	0	0	0	0	0	0	2.756	0	0	0	0	0	0	0	0	152.4	0	0	0	1.143	0
17	0	0	0	0	0.586	0	140	0	0.255	0	0	0.289	0.12	0	0	0	0	0	0	0	0	0.317	0	0
18	0	0	0	179.7	0	0	0	0	0	0	0	0	0.03	0	0	0	0	0	0	0.047	0	0	0	0
19	0	0	181.8	0	0	0	0	0	0	0	0	0	0	0	0	0	0	0	0	0	0	0.1	0	0
20	1.402	0	0.181	0	0	0	0.747	0	0	0.282	0.292	0	0.949	0.315	0	0	0.464	0	0	0.773	0	0.105	0	0.164
21	0	0	0	0	0	0	0	0	0	0	0	0	0	0	0	0	0	0	0	0	0	0	0	0
22	0	2.597	0	0	0	0	0	111.6	0	0	0	0	0	0	0	0	0	0	0	0	0	0	0	0
23	0	0	0.689	0.253	0	0.331	0	0	0.345	0	0	0	0	0	0	0	0	0	0.067	0.086	0	0.154	0	0.225
24	0	0.011	0	0	11.79	0	0	0	0	0	0	0	0.076	0	2.254	0	0	0	0	0	0	0	0	0
25	0.698	0	0	0.284	0	0	0	0	0	0	0	0	0	0	0	0	0	0	0	0.105	0	0	0	0.255
26	0	0	0	0	0	0	0	0	0	0.407	0	0	0.287	0	0	0	0	0	0	0.114	0	0	0	0
27	0	0	0	0	0	0	0	0	0	0	0.419	147.4	0	0	0	0	0	0	0	0	0	0	0	0
28	0	0	0	0	0	0	0	0	0	0	0	0	0	0	0	128.2	6.521	0	0	0	0	0	0	0
29	0	0	0.302	0.312	0	0.396	0	0	0.411	0	0.434	0	0	0	0	0	0	0	0.099	0	0	0	0	0
30	0	0	0	0	0	0	0	0	127.4	0	0	0	0	0	0	0	0	0	0	0	0	0	0	0
31	0	0	0	0	0	0	0	0	0.451	0	0	0	0	0	0	0	0	0	0	0.147	0	0	0	0
32	0	0	0.344	0	0	0	0	0.457	0	0	0	0	0.115	0	0	0	0	0	0	0.151	0	0	0	0
33	0.795	0	0	0	0	0.448	1.174	0	0	0	0	0	0.119	0	0	0	0	0	0	0.155	0	0	0	0
34	0	0	0.356	0	0	0	0	0	0	0.482	0	0	0.394	0	0.614	0	0	0	0	0.16	0	0	0	0
35	0	0	0	0	0	0	0	0.477	0	0	97.96	0.52	0.127	0	0	0	0	0	0.137	0	0	0	0	0
36	0	0	0.368	0	0	0	0	0	0	0	0	0	0	0	0	0	0	0	0	0.168	0	0	0	0.346
37	0.826	0	0	0	0	0	0	0	0	0	0.516	0	0	0	0	0	0.715	0	0	1.015	0	0.265	0	0
38	0	0	0	0	0	0	0	0	0	0	0	0	0.139	0	0	0	0	0	0	0.178	0	0	0	0
39	0	0	0	0	0	0	0	0	0	0	0	0	0	0	0	0	0	0	0	0.183	0	0	0	0
40	0	0	0	0	0	0	0	0	0	0	0	0	0.91	0	0	0	0	0	0	0.559	0	0	0	0
41	0	0	0	0.428	0	0	0	0	0	0	0	0	0.497	0	0	0	0	0	0	0	0.298	0	0	0.394
42	0	0	0.417	0	0	0	0.533	0	0	0	0.563	0	0	0	0	0.686	0	0	0	0	0	0	0	0
43	0	0	0	0	0	0	0	0	0	0	0.563	0	0.497	0	0	0	0	0	0	0.205	0	0	0	0
44	0.887	0	0	0	0	0.529	0	0	0	0	0	0	0.168	0	0	0	0	0.909	0	1.168	0	0.31	0	0
45	0	0	0	1.141	0	0.538	0.55	0	0	0.567	0	0	0.524	0	0.704	0	0	0	0	0	0	0	0	0
46	2.095	0	0	0	0	0	0	0	0	0	0.58	0	0	0	0	0	0	0	0	0	0	0	0	0
47	0	0	1.117	0	0	0	0	0	0	0	0	0	0.174	0	0	0	0	0	0	0	0	0	0	0.409
48	0.896	0	0	0	0	0	0	0	0	0	0	0	0	0	0	0	0	0	0	0	0	0	0	0
49	0	0	0	0	0	0	0	0	0	0	0	0	0	0	0	0	0	0	0	0	0	0.34	0	71.01
50	0	0	0	0	0	0	0	0	0.581	0	2.559	0.626	0.192	0	0	0	0	0	0	0	0	0.913	0	0
51	0	0	0	0	0	0	0	0	0	0.635	0	0	0.636	0	0	0	0	0.993	0	0	0	0	0	0
52	0	0	0	0	0	0	0	0	0	0	0	0	0.219	0	0	0	0	0	0	0	0	0.375	0	0
53	0	0	0	0	0	0	0	0.633	0	0	0	0	0	0	0	0	78.92	0	0	0	0	0	0	0
54	0	0	0	0.53	0	0	0.64	0	0	0	0.671	0	0	0	0	0	0	0	0	1.454	0	0	0	0
55	0	0	0	0	0	0	0	0	0	0	0	0	0	0	0	0	0	80.11	0	0	0	0.394	0	0

P-value (-$\log_{10}$)

0 10

c

Classification by all 55 modules

```
        OOB estimate of  error rate: 20.45%
Confusion matrix:
    C1 C10 C2 C3 C4 C5 C6 C7 C8 C9 class.error
C1  42   0  2  1  0  1  0  0  0  0  0.08695652
C10  0  26  0  0  1  0  0  0  2  3  0.18750000
C2   0   0 34  0  0  0  1  0  1  0  0.05555556
C3   5   0  0 17  0  1  1  0  0  0  0.29166667
C4   1   3  1  2  6  3  1  0  2  5  0.75000000
C5   0   0  4  0  0 57  0  0  0  0  0.06557377
C6   0   0  1  0  0  2 39  0  0  0  0.07142857
C7   1   4  1  0  0  0  0 12  5  3  0.53846154
C8   1   0  2  0  0  0  2  2 47  6  0.21666667
C9   0   3  0  0  1  0  0  3  3 35  0.22222222
```

Classification by selected 9 modules

```
        OOB estimate of  error rate: 5.63%
Confusion matrix:
   C1 C2 C3 C4 C5 C6 C7 class.error
C1 77  2  1  0  0  0  0  0.03750000
C2  2 62  1  0  0  0  0  0.04615385
C3  1  0 47  0  0  0  0  0.02083333
C4  1  1  0 35  1  1  1  0.12500000
C5  0  0  2  0 33  0  0  0.05714286
C6  0  0  0  3  0 53  2  0.08620690
C7  0  0  1  1  0  1 62  0.04615385
```

d

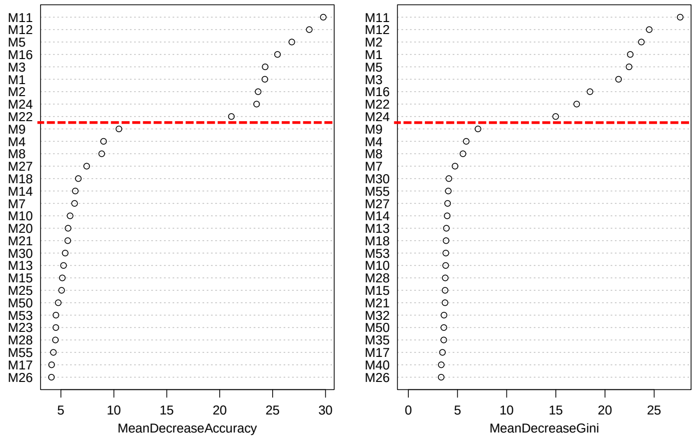

b

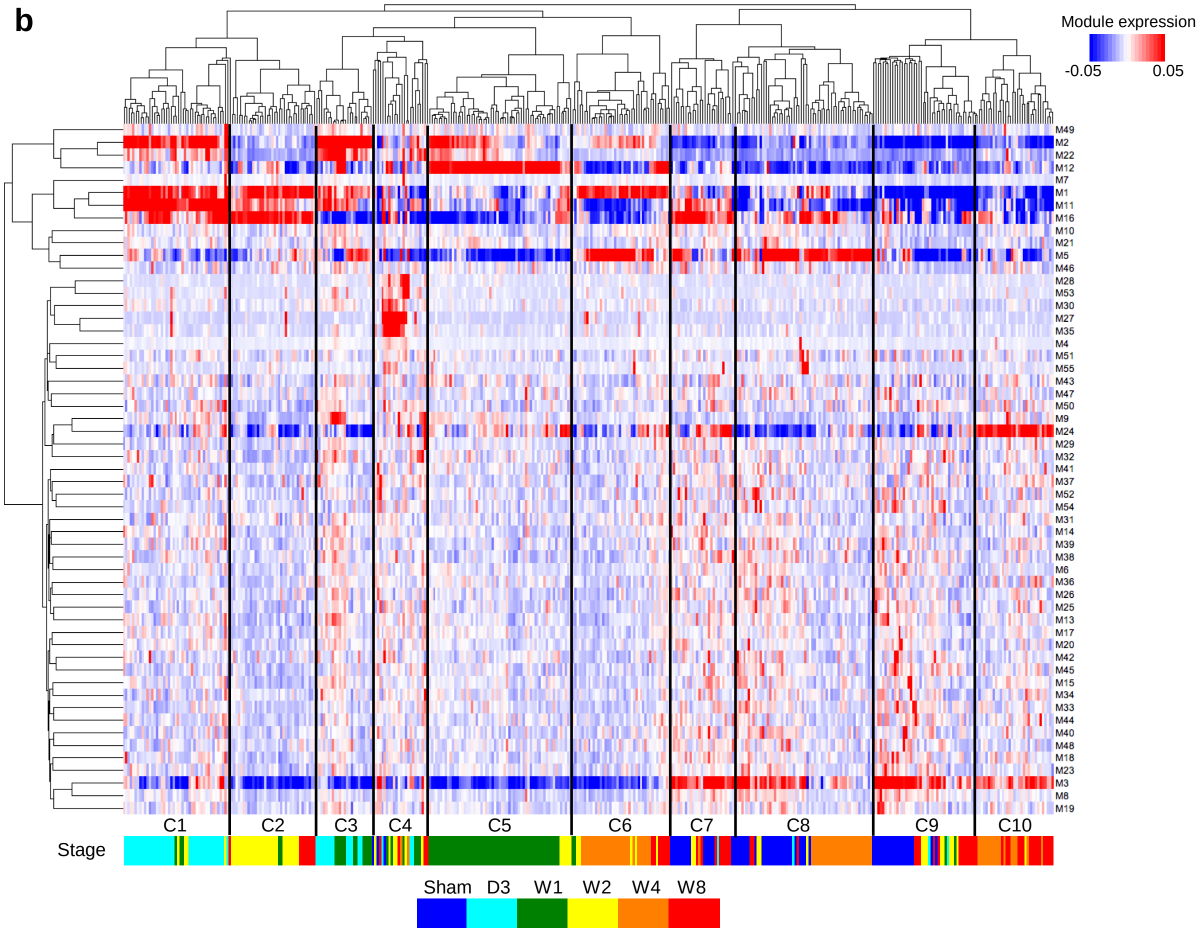

(legend on next page)

Supplementary Figure 6. Co-expression module-based clustering.

(**a**) Cross-tabulation table of the modules obtained using expression values from Bowtie (columns) and Tophat (rows). The table is colored by $-\log_{10}(P\text{-value})$, obtained with Fisher's exact test, according to the color legend below the table. The selected 9 modules were recovered in the gene modules obtained using Tophat.

(**b**) Hierarchical clustering of the expression of all 55 modules detected by weighted gene co-expression network analysis (WGCNA). The colored bar below the heatmap indicates the time when the cardiomyocytes were extracted.

(**c**) Representative estimated error rates of classifications by all 55 modules (top) and 9 selected modules (bottom).

(**d**) Mean decrease accuracy and mean decrease Gini (sorted decreasingly from top to bottom) of attributes as assigned by the Random Forest classifier. The red lines indicate the significant thresholds used in this study.

Supplementary Figure 7

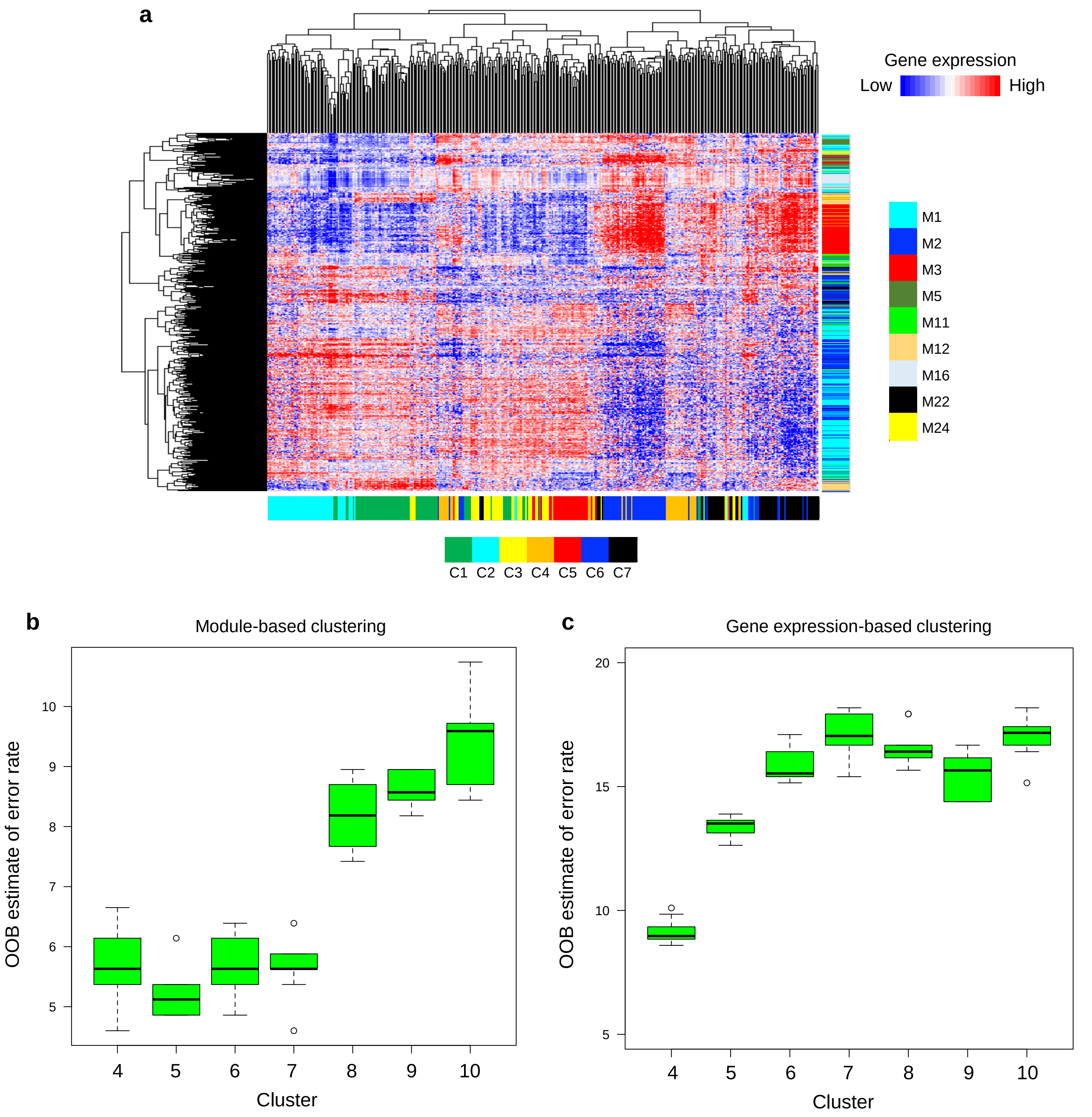

Supplementary Figure 7. Assessment of classification accuracy.

(**a**) Hierarchical clustering using the expression levels of the genes assigned to the 9 modules (M1, M2, M3, M5, M11, M12, M16, M22, and M24).

(**b**,**c**) Boxplot showing changes in the out-of-bag (OOB) error rates at the indicated numbers of clusters in **Figure 1d** (module-based clustering, **b**) and in **Supplementary Figure 7a** (gene expression-based clustering, **c**). Assessment of classification accuracy using Random Forests was performed 10 times. Boxplots show medians (central bars), the 1st to 3rd quartile range (boxes), other data out to 1.5 the interquartile range (whiskers), and outliers (dots).

Supplementary Figure 8

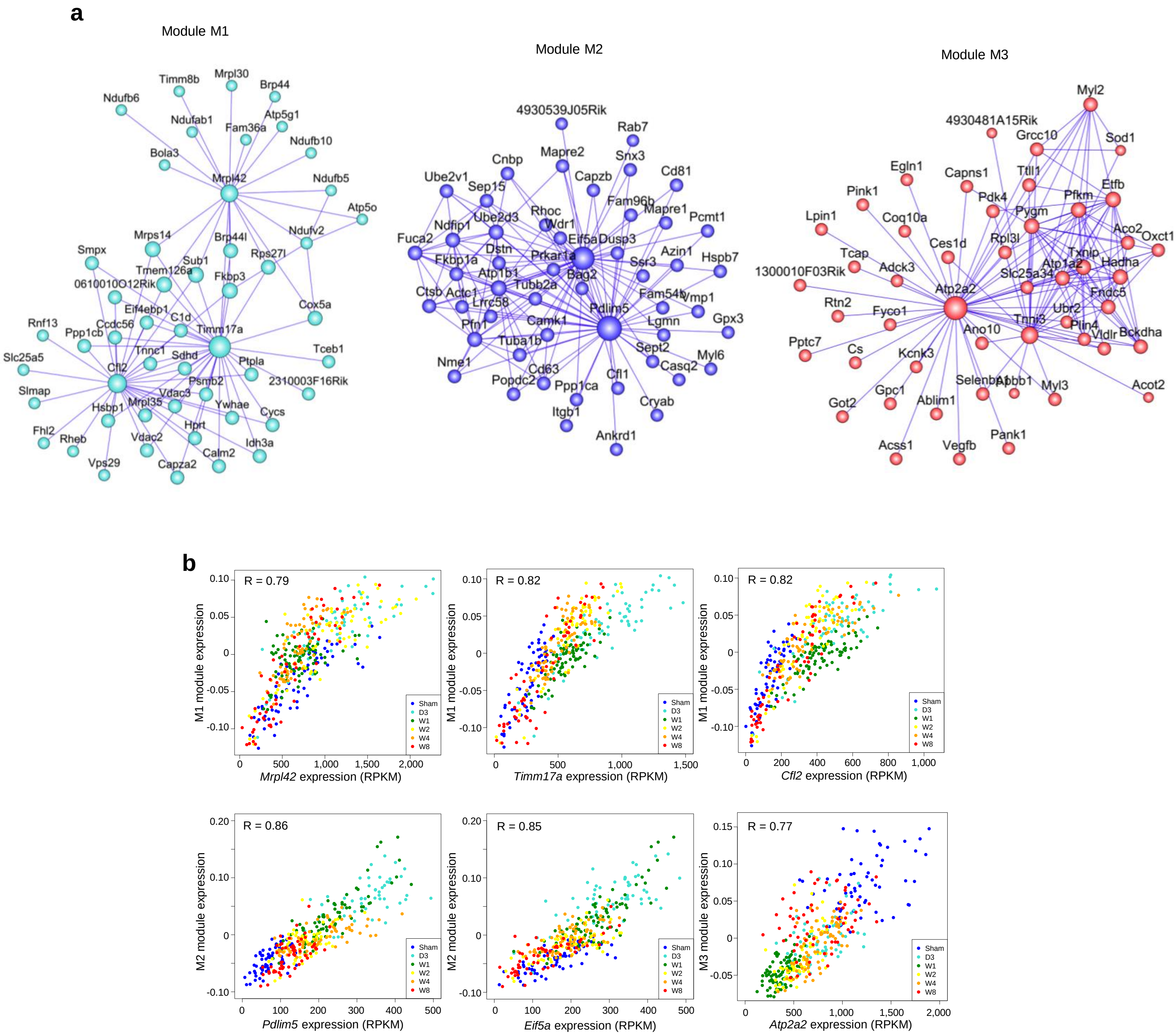

Supplementary Figure 8. Hub gene network analysis.
(**a**) Hub gene networks of M1, M2, and M3. The size of the dots represents node centrality.
(**b**) Scatter plot showing the correlations between module expression and the expression of hub genes. Samples are colored according to when the cardiomyocytes were extracted.

Supplementary Figure 9

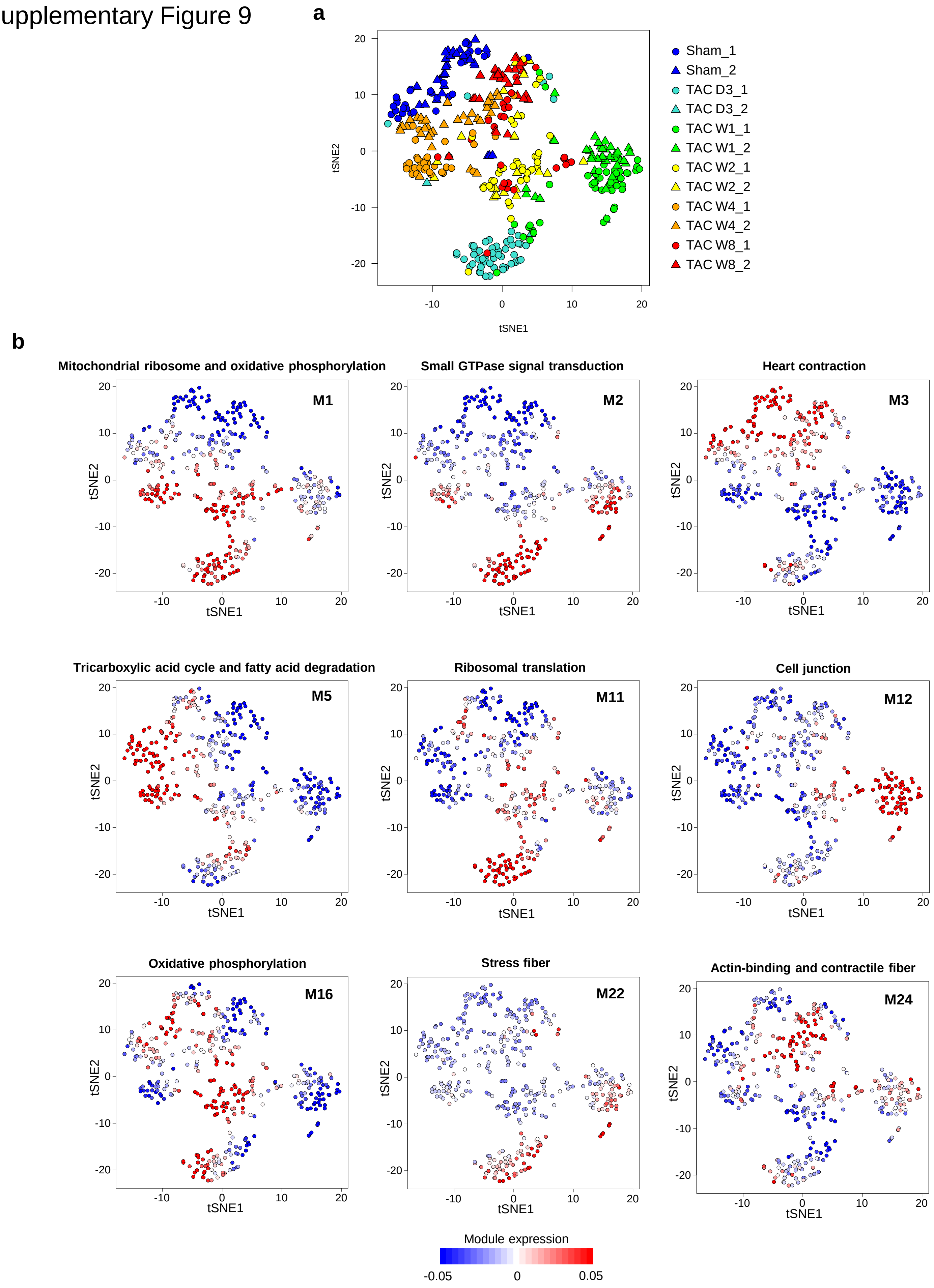

Supplementary Figure 9. Module expression on a t-SNE map.
(**a**) *t*-distributed stochastic neighbor embedding (t-SNE) plot (**Figure 1g**) colored by batch.
(**b**) t-SNE plots colored by the expression levels of each module.

Supplementary Figure 10

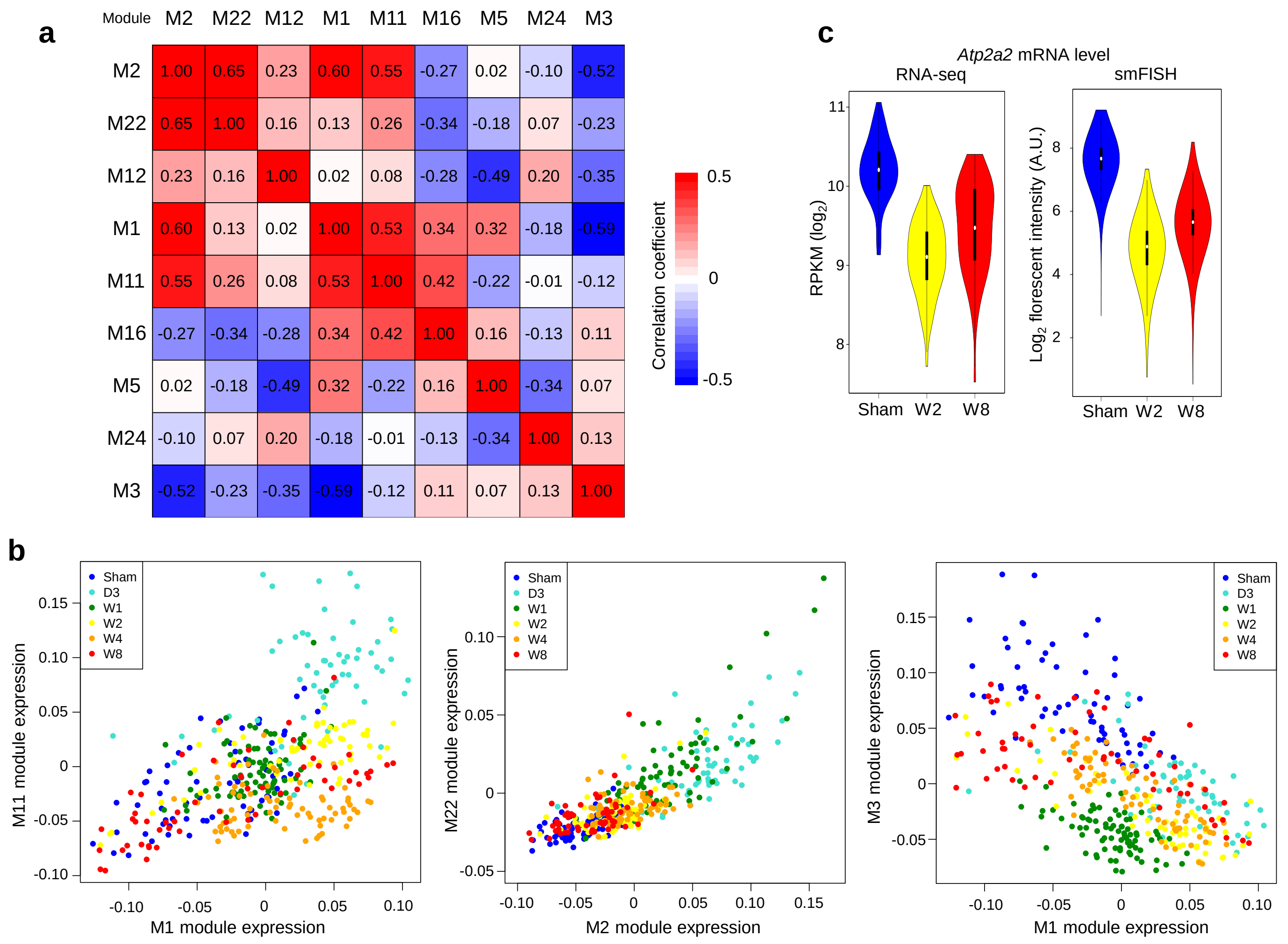

Supplementary Figure 10. Module-to-module correlation analysis.
(**a**) Pearson correlation matrix showing the correlations between modules.
(**b**) Scatter plots showing the correlations between modules.
(**c**) Violin plot showing the dynamics of single-cell *Atp2a2* mRNA expression levels in cardiomyocytes detected by single-cell RNA-seq (left) and smFISH (right).

Supplementary Figure 11

a

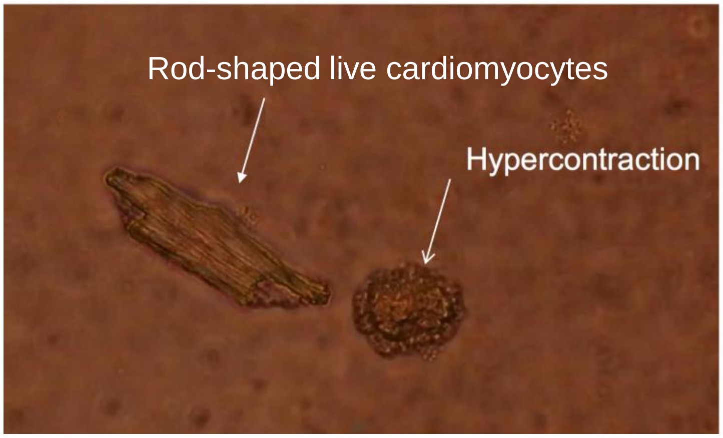

b

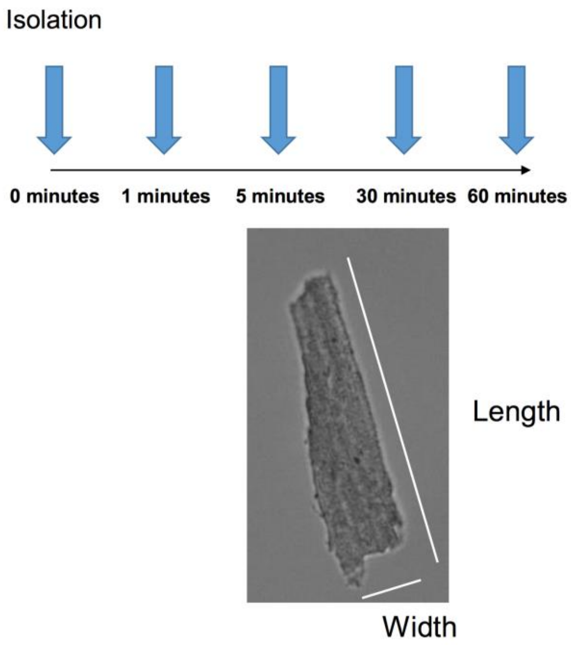

c

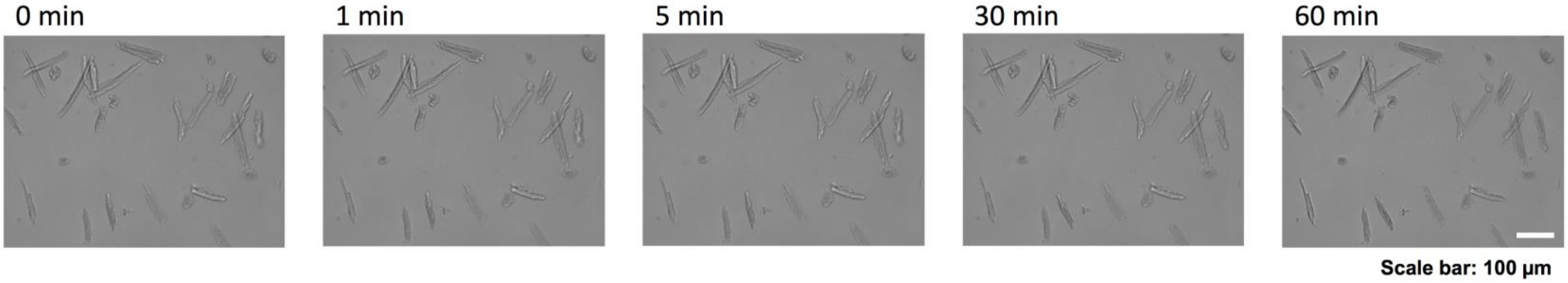

d

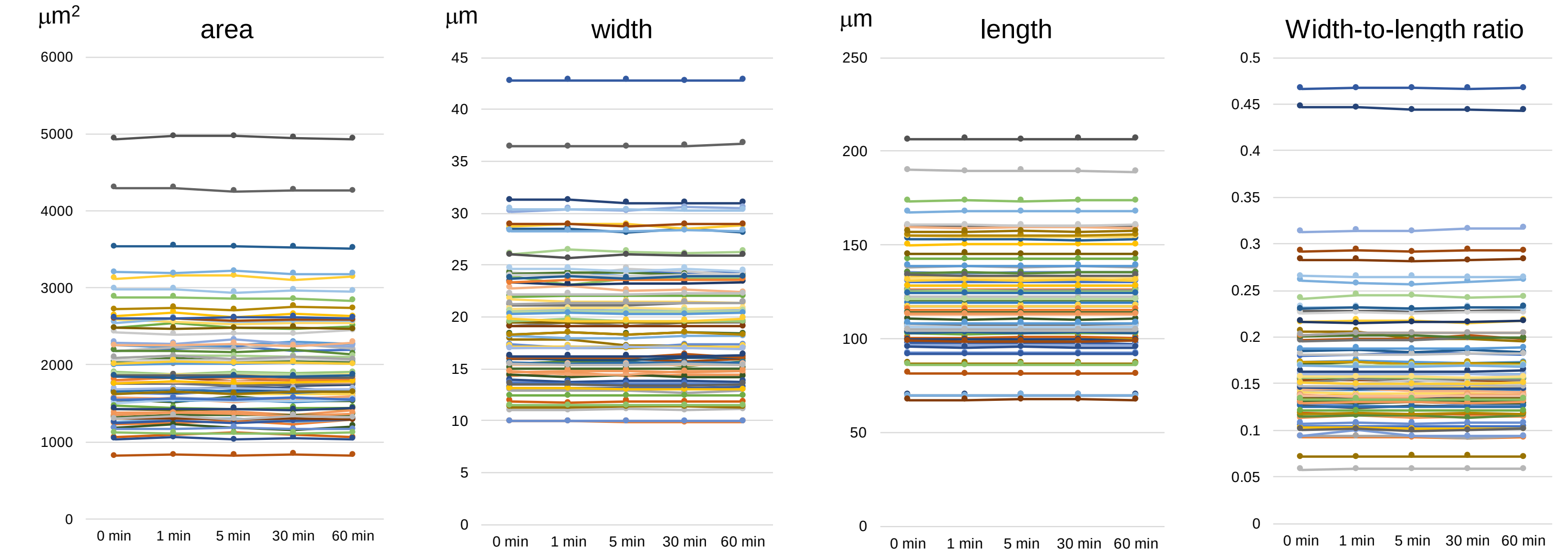

e

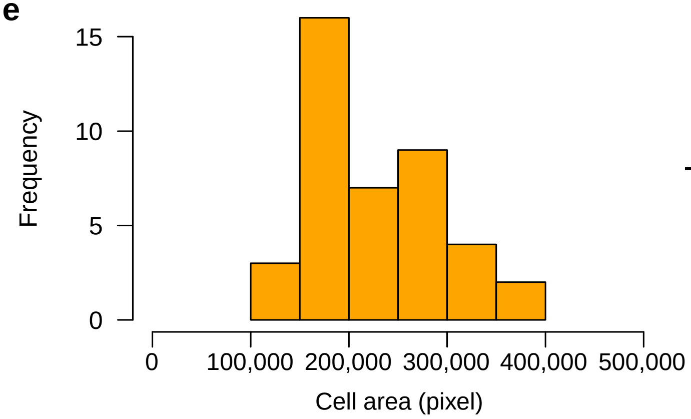

f

Normalized by an endogenous control

GO term	Count	*P*-value
Mitochondrion (GO:0005739)	72	3.50E-20
Mitochondrial inner membrane (GO:0005743)	22	2.00E-08
Cell redox redox homeostasis (GO:0045454)	9	1.60E-06
Mitochondrial ribosome (GO:0005761)	5	2.50E-04

Supplementary Figure 11. Preliminary morphological assessment of isolated cardiomyocytes
(**a**) Morphological differences between rod-shaped live cardiomyocytes and cardiomyocytes causing hypercontraction.
(**b**) Overview of the assessment of the morphological dynamics of cardiomyocytes after isolation.
(**c**) Representative time-lapse images of cardiomyocytes.
(**d**) Line graph showing the dynamics of morphological parameters (cell area, width, length, and width-to-length ratio) after isolation.
(**e**) Histogram of the area of cells used in **Fig. 3**. Morphological parameters were maintained after isolation.
(**f**) List of the most enriched GO terms in the top 300 correlated genes with cell area. Gene expression values were normalized by an endogenous control (*Tnnt2*).

Supplementary Figure 12

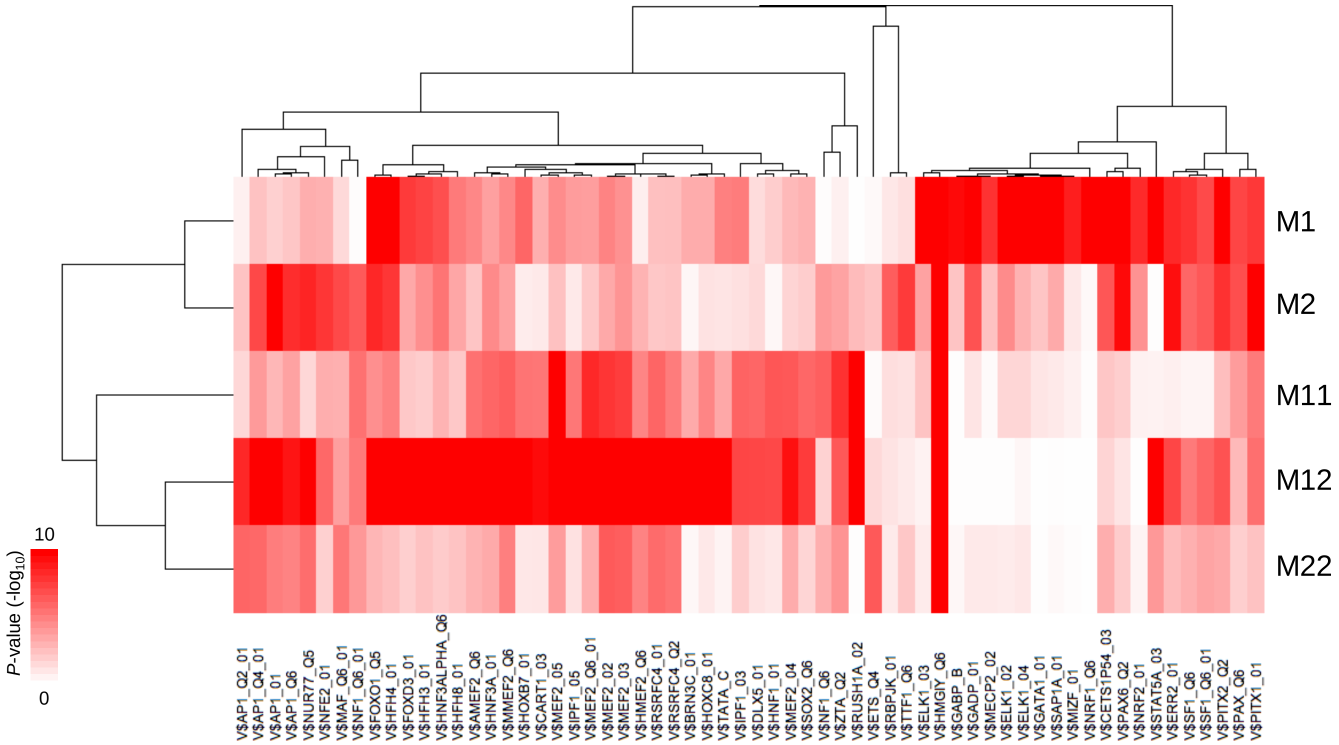

Supplementary Figure 12. Correlation between modules and hypertrophy-related regulatory factors.
Detailed presentation of hierarchical clustering of the enrichment of transcription factor recognition motifs in **Fig. 3l**. The top 10 significantly enriched motifs for each module are selected.

Supplementary Figure 13

a

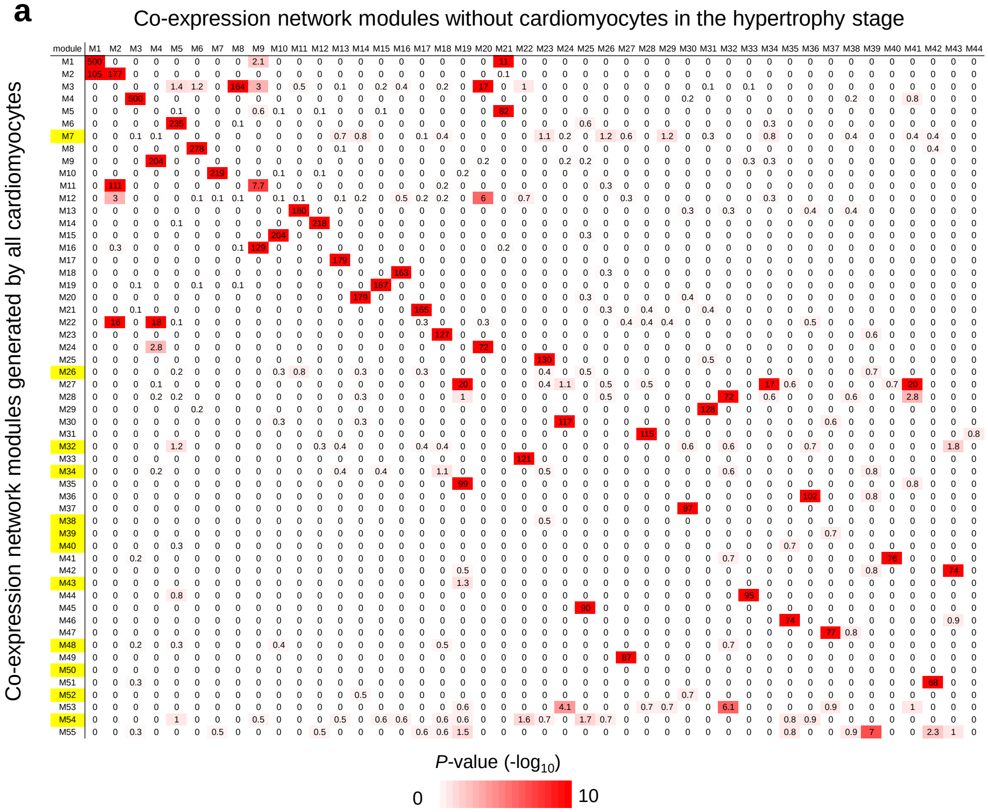

b

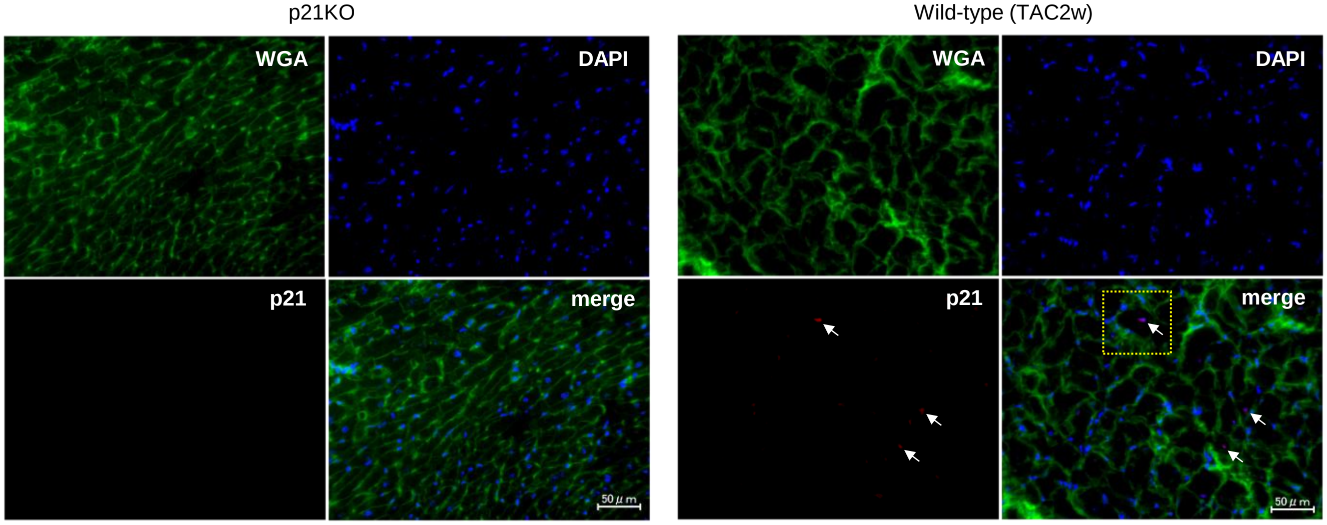

c

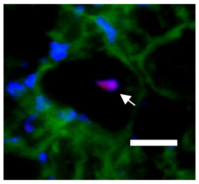

d

Response to oxidative stress

Module	M1	M2	M3	M5	M11	M12	M16	M22	M24
enrichment *P*-value	2.05	0.00	0.00	2.25	0.00	0.00	0.00	0.00	0.00

e

Genes	Prdx1	Prdx3	Ndufs2	Sod2	Glrx2	Nqo1	Gpx1	Prdx6	Cat	Lias
Correlation	0.44	0.38	0.35	0.30	0.23	0.22	0.22	0.18	0.15	0.10

f

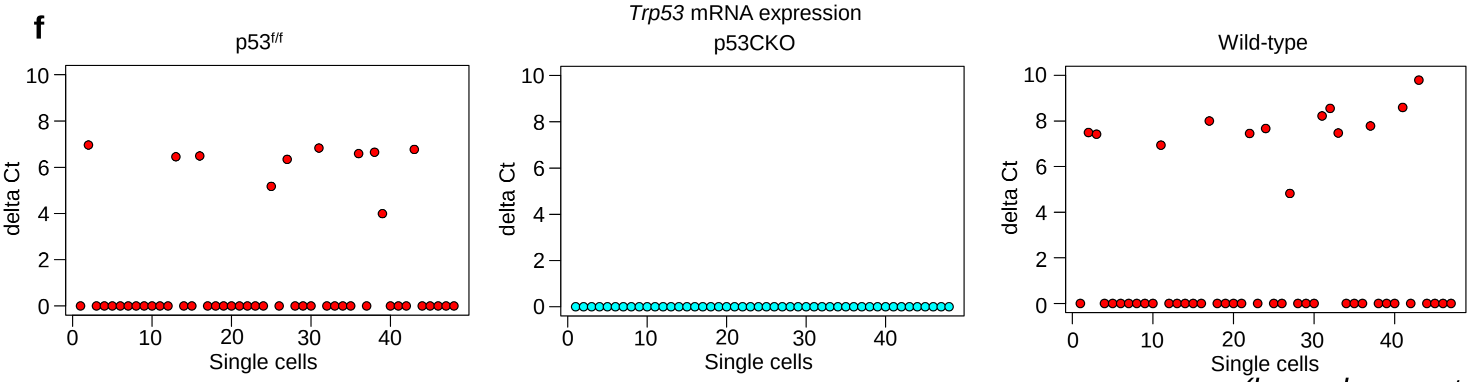

(legend on next page)

Supplementary Figure 13. Identification and assessment of hypertrophy stage-specific modules.
(**a**) Cross-tabulation table of modules obtained using all cardiomyocytes (columns) and modules obtained using cardiomyocytes without the hypertrophy stage (at 1 and 2 weeks after pressure overload) (rows). The table is colored by $-\log_{10}$(*P*-value), obtained with Fisher's exact test, according to the color legend below the table. Modules colored yellow are hypertrophic stage-specific (all module enrichment *P*-values were >1e-5).
(**b**) Assessment of p21 immunostaining in cardiomyocytes. p21 protein was observed in a small fraction of cardiomyocytes of wild-type mice at 2 weeks after TAC, but not in cardiomyocytes of p21 knockout mice. WGA and DAPI are used as markers of the plasma membrane and nucleus, respectively. Scale bar, 50 μm. The arrows indicate the nuclei of p21-positive cardiomyocytes.
(**c**) Representative higher magnification image of p21-positive cardiomyocytes (area of dashed quadrangle in **b**). Scale bar, 20 μm.
(**d**) Gene ontology analysis showing the enrichment of genes involved in the oxidative stress response.
(**e**) Correlation coefficient between cell area and the expression of genes involved in the oxidative stress response.
(**f**) Single-cell quantitative real-time polymerase chain reaction of *Trp53* mRNA (encoding p53) in cardiomyocytes from $p53^{flox/flox}$ ($p53^{f/f}$), p53CKO, and wild-type mice at 2 weeks after pressure overload.

Supplementary Figure 14

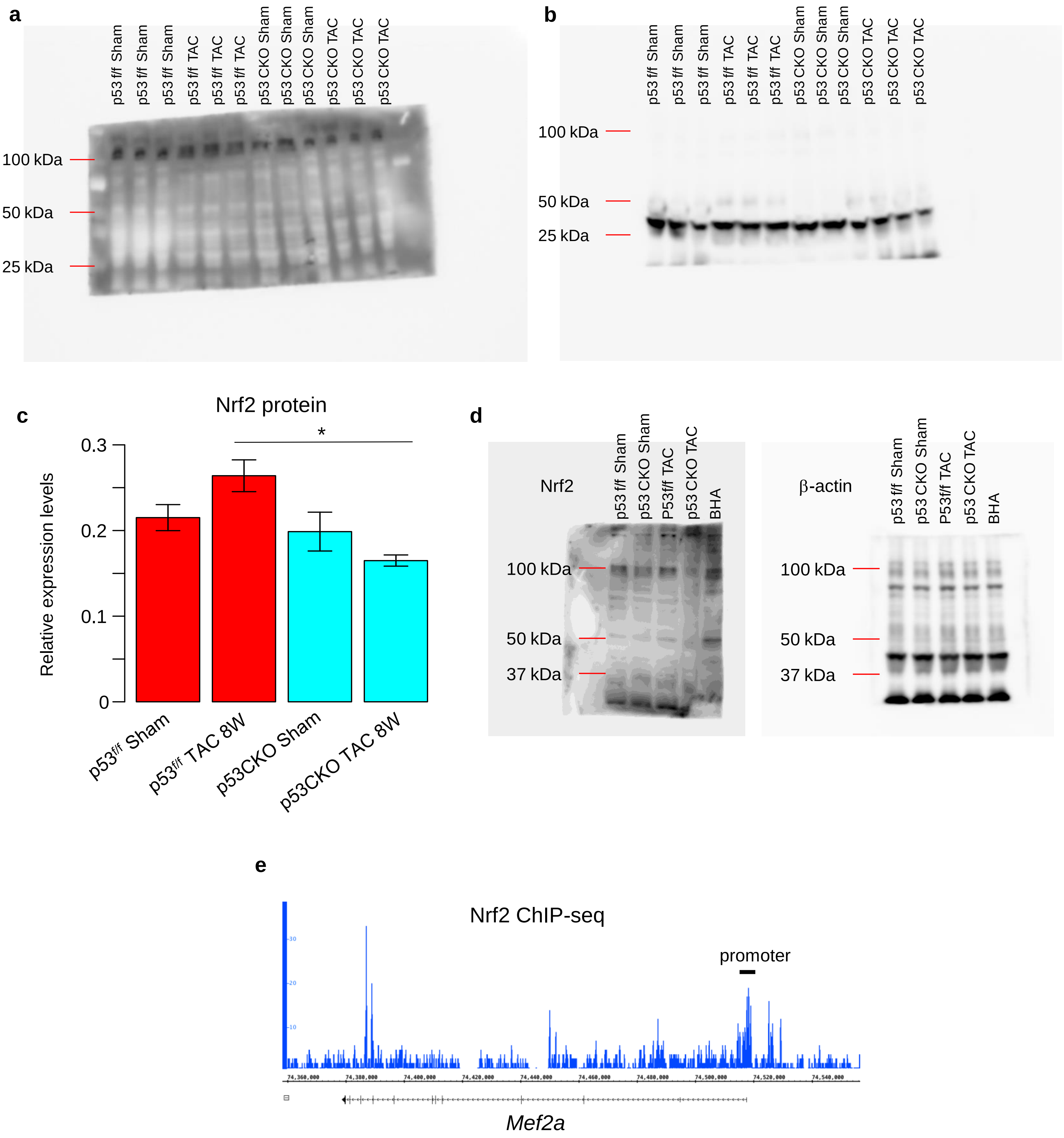

Supplementary Figure 14. p53-Nrf2-Mef2a axis in failing cardiomyocytes.
(**a**,**b**) Western blot analysis of heart tissues using antibodies against Nrf2 (**a**) and β-actin (**b**) (*n* = 3 each).
(**c**) Densitometric quantification of Nrf2 protein expression levels normalized by β-actin expression levels. Data are represented as mean and standard error of the mean (*n* = 3 each). Asterisks indicate statistical significance ($P < 0.01$, two-tailed unpaired *t*-test).
(**d**) Uncropped images of the blots for **Fig. 6e**.
(**e**) Public Nrf2 ChIP-seq data of macrophages (GSE72964) showing Nrf2 binding to the *Mef2a* promoter.

Supplementary Figure 15

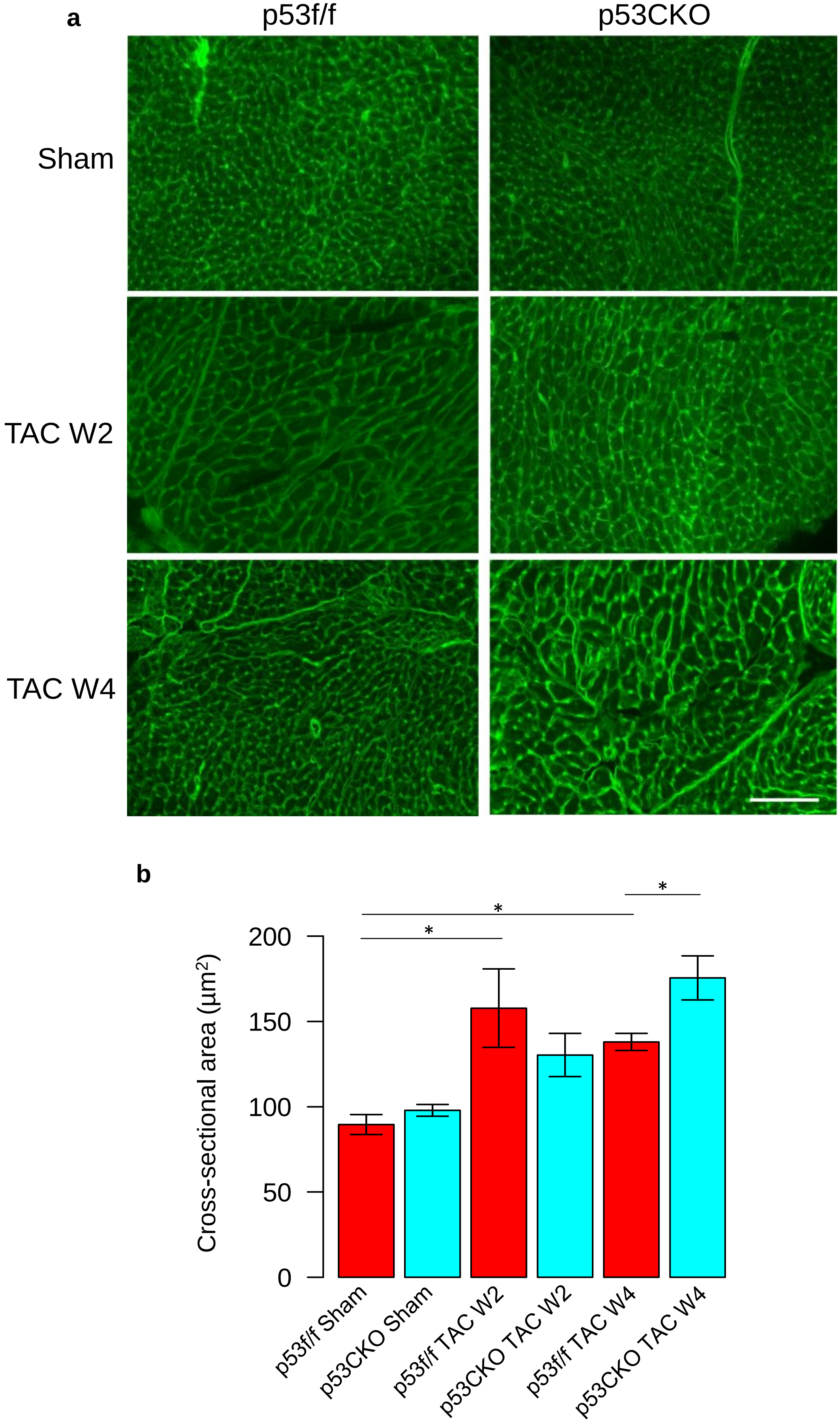

Supplementary Figure 15. Measurement of cardiomyocyte cross-sectional area.
(**a**) Representative images of wheat germ agglutinin-stained cardiac sections.
(**b**) Bar plots showing the quantitative measurement of cardiomyocyte cross-sectional area. Data are represented as the mean and standard error of the mean (n = 6). Asterisks indicate statistical significance ($P < 0.05$, two-tailed unpaired t-test).

Supplementary Figure 16

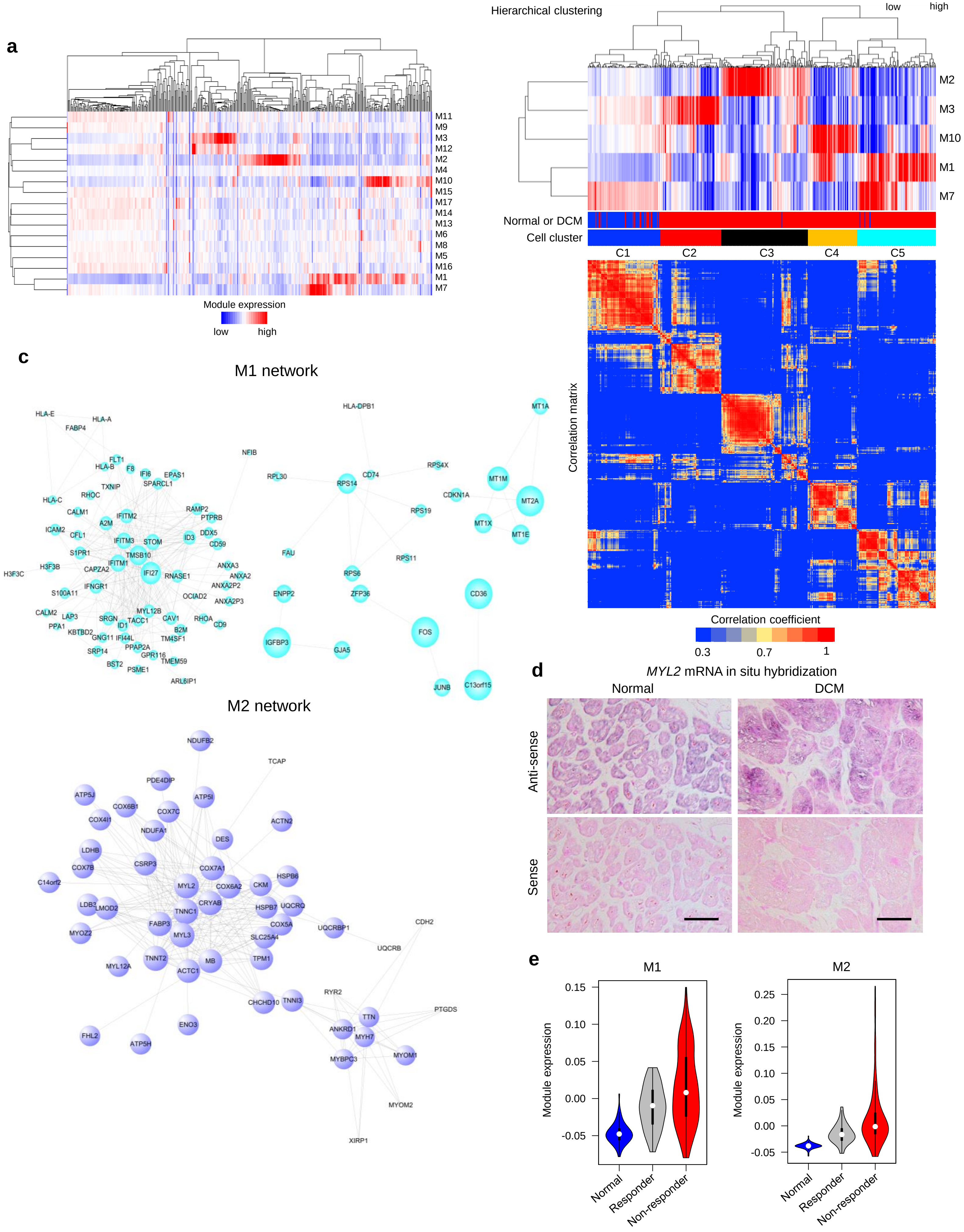

Supplementary Figure 16. Distinct gene programs and their pathogenicity in human cardiomyocytes.
(**a**) Hierarchical clustering of human cardiomyocytes using the expression of all 17 modules detected by WGCNA.
(**b**) Unsupervised co-expression module-based clustering classifying all cardiomyocytes (n = 411) into 5 cell clusters (C1–C5). The colored bar below the heatmap indicates the origin of each cardiomyocyte (normal subjects [blue] or dilated cardiomyopathy [DCM] patients [red]) and the cell clusters. The correlation matrix is also indicated.
(**c**) The co-expression networks of M1 and M2. The size of the dots represents node centrality.
(**d**) Representative images of *MYL2* mRNA *in situ* hybridization analysis of the heart from normal subjects and DCM patients. The anti-sense probe specifically hybridized with *MYL2* mRNA, while the sense probe was used as a negative control. Scale bar, 50 μm.
(**e**) Violin plot showing the distribution of module expression.
