# Supplementary material for: Cardiomyocyte gene programs encoding morphological and functional signatures in cardiac hypertrophy and failure
Source: Nat Commun. 2018 Oct 30;9:4435. doi: 10.1038/s41467-018-06639-7 (PMC6207673; doi:10.1038/s41467-018-06639-7)
Supplement: Supplementary file 3 — Description of Additional Supplementary Files [file 41467_2018_6639_MOESM3_ESM.pdf]

Description of Additional Supplementary Files

File Name: Supplementary Data 1
Description: Sequencing quality data of mouse cardiomyocytes.

File Name: Supplementary Data 2
Description: Sequencing quality data of human cardiomyocytes.

File Name: Supplementary Data 3
Description: Myh7 and Atp2a2 mRNA smFISH intensity at week 8.

File Name: Supplementary Data 4
Description: Module annotation in mouse cardiomyocytes.

File Name: Supplementary Data 5
Description: Atp2a2 mRNA smFISH dynamics.

File Name: Supplementary Data 6
Description: Integrative analysis of single-cell morphology and gene expression of TAC W1 cardiomyocytes.

File Name: Supplementary Data 7
Description: Cdkn1a mRNA smFISH dynamics.

File Name: Supplementary Data 8
Description: Raw data of Figure 5b, Figure 6f, and Supplementary Figure 14c.

File Name: Supplementary Data 9
Description: Raw data of Supplementary Figure 15b.

File Name: Supplementary Data 10
Description: Module annotation in human cardiomyocytes.

File Name: Supplementary Data 11
Description: Primer sequences.
